# Monte Carlo‐based lung cancer treatment planning incorporating PET‐defined target volumes

**DOI:** 10.1120/jacmp.v6i4.2156

**Published:** 2005-11-22

**Authors:** Indrin J. Chetty, Shaneli Fernando, Marc L. Kessler, Daniel L. Mcshan, Cassandra Brooks, Randall K. Ten Haken, Feng‐Ming (Spring) Kong

**Affiliations:** ^1^ University of Michigan Department of Radiation Oncology 1500 E. Medical Center Dr., UH‐B2‐C438 Ann Arbor Michigan 48109‐0010 U.S.A.

**Keywords:** PET imaging, lung cancer, Monte Carlo, target segmentation

## Abstract

Despite the well‐known benefits of positron emission tomography (PET) imaging in lung cancer diagnosis and staging, the poor spatial resolution of PET has limited its use in radiotherapy planning. Methods used for segmenting tumor from normal tissue, such as threshold boundaries using a fraction of the standardized uptake value (SUV), are subject to uncertainties. The issue of respiratory motion in the thorax confounds the problem of accurate target definition. In this work, we evaluate how changing the PET‐defined target volume by varying the threshold value in the segmentation process impacts target and normal lung tissue doses. For each of eight lung cancer patients we retrospectively generated multiple PET‐target volumes; each target volume corresponds to those voxels with intensities above a given threshold level, defined by a percentage of the maximum voxel intensity. PET‐defined targets were compared to those from CT; CT targets comprise a composite volume generated from breath‐hold inhale and exhale datasets; the CT dataset therefore also includes the extents of tumor motion. Treatment plans using Monte Carlo dose calculation were generated for all targets; the dose uniformity was approximately 100±5% within the internal target volume (ITV) (formed by a uniform 8‐mm expansion of the composite gross target volume (GTV)). In all cases differences were observed in the generalized equivalent uniform doses (gEUDs) to the targets and in the mean lung doses (MLDs) and normal tissue complication probabilities (NTCPs) to the normal lung tissues. The magnitudes of the dose differences were found to depend on the target volume, location, and amount of irradiated normal lung tissue, and in many instances were clinically meaningful (greater than a single 2 Gy fraction). For those patients studied, results indicate that accurate dosimetry using PET volumes is highly dependent on accurate target segmentation. Further study with correlation to clinical outcome will be helpful in determining how to apply these various PET and CT volumes in treatment planning, to potentially improve local tumor control and reduce normal tissue toxicities.

PACS number: 87.52.Tf

## I. INTRODUCTION

The use of positron emission tomography (PET) imaging for the diagnosis and staging of malignancies has important implications in clinical radiation therapy. The ability of F‐18 labeled deoxyglucose (FDG), for example, to noninvasively assess glucose metabolism provides a distinct advantage over nonfunctional imaging modalities, such as CT.^(^ ^1^ ^–^ ^3^ ^)^ With respect to lung cancer radiotherapy planning, over the past five years investigators have begun to take advangtage of the improved ability of PET to delineate target volumes. More specifically, studies have focused on the following areas: (1) assessment of differences in PET‐ versus CT‐defined target volumes and reduction in intra‐observer defined target volumes when incorporating PET imaging into treatment planning,^(^ ^4^ ^–^ ^6^ ^)^ (2) differences in treatment‐planning metrics resulting from the inclusion of PET‐defined targets,^(7)^ and (3) incorporating functional information derived from PET imaging to optimize radiotherapy dose distributions.^(^ ^8^ ^,^ ^9^ ^)^

Despite the well‐known benefits of PET imaging in radiotherapy planning, the poor spatial resolution associated with this modality has limited its use in the clinical setting. Methods used for segmenting tumor from normal tissue, such as threshold boundaries using a fraction of the standardized uptake value (SUV), are subject to uncertainties. Although phantom studies have been performed to quantify segmentation threshold as a function of target size,^(^ ^10^ ^,^ ^11^ ^)^ such phantom studies are unlikely to accurately represent true clinical conditions where organ motion becomes a confounding factor.^(12)^ PET is a “slow” scan, which is acquired over all phases of breathing; the PET positive volume therefore includes motion of the tumor. The presence of organ motion in lung cancer in conjunction with poor spatial resolution results in uncertainties in the segmentation of tumor from normal tissue. In this work, we investigate the dosimetric consequences of uncertainties in the PET target definition resulting from the segmentation process in lung cancer radiotherapy treatment planning.

## II. METHODS

### A. CT and PET imaging, target definition, and image fusion

CT and PET images were acquired for patients being treated for lung cancer on an Institutional Review Board‐approved imaging protocol. CT scanning was performed with a GE CT/i scanner at each of three phases of breathing: quiet expiration breath‐hold, quiet inspiration breath‐hold, and free breathing. CT slice thicknesses were 5 mm. Gross tumor volumes (GTVs) were contoured on the breath‐hold exhale and inhale CT datasets, and the contours were combined to produce a 3D composite GTV. The composite GTV was further expanded uniformly by 8 mm in 3D to generate an internal target volume (ITV). GTVs for all eight patients were delineated by the same physician. Clinical target volumes (CTVs) were also generated from uniform (5 mm) expansion of the GTVs. However, CTV analysis was not part of this work; the main intent here was to evaluate dosimetric differences resulting from the use of CT‐ versus PET‐defined target volumes.

PET images were acquired with Siemens (Biograph or ECAT) scanners. According to the imaging protocol, PET images were acquired no later than one week from the time of the CT image acquisition. Although the CT and PET scans were obtained on different scanners, the CT treatment position was reproduced in the PET scanner using the same custom immobilization and a flat surface couch attachment, ultimately to facilitate accurate PET/CT registration. For target volume definition, attenuation‐corrected emission PET images were registered with the breath‐hold exhale CT dataset, which is the reference dataset for treatment‐planning purposes. The breath‐hold exhale CT dataset serves as the reference dataset in our routine clinical lung cancer planning for the following reasons: (1) approximately 70% of the time‐weighted breathing cycle is spent at exhale, and (2) the exhale position provides the most conservative estimate of the normal tissue complication probability as the volume of irradiated normal lung is minimized. PET/CT registration was accomplished using a thin‐plate spline transformation model (TPS)^(13)^ available within our in‐house treatment‐planning system, UMPlan. In this method, control points are placed manually on the image datasets and are algorithmically manipulated to produce an optimal transformation corresponding to the maximum mutual information between the two image datasets. This approach was used previously^(^ ^14^ ^,^ ^15^ ^)^ to map, with sufficient accuracy, the lungs or liver between two CT scans acquired at different breathing phases for the same patient. It should also be noted that the control points in this study were placed in the organs of interest—the tumor and normal lungs—in order to optimize registration using local measures.^(16)^ PET GTVs were contoured using a segmentation method based on a fraction of the maximum voxel intensity (a surrogate for the maximum standardized uptake value (SUV)) in the PET images; that is, the GTV was formed by considering all voxels above a given percentage of the maximum intensity voxel. The maximum voxel intensity corresponded to the single voxel with the highest intensity within the PET dataset. Tumor and mediastinal lymph nodes for centrally located tumors were included within the target definition. In PET‐positive regions within the mediastinum, the medial border of the contour was manually edited using CT to exclude the mediastinal blood pool, thereby minimizing the influence of background activity in the target definition. Multiple PET GTVs were generated with each target corresponding to a different segmentation threshold value; values in this study ranged from 10% to 50% of the maximum intensity. In a previous study, volumetric differences between the various PET‐defined GTVs relative to the CT‐defined composite GTV and ITV^(17)^ were quantified.

**Figure 1:**
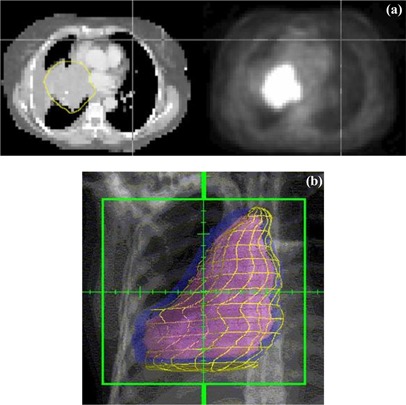
(a). CT image (left) and corresponding PET image (right) showing the good spatial correlation between the comp_GTV (CT‐defined) in the yellow contour and the region of tumor 18‐FDG uptake, based on a 20%‐of‐maximum segmentation threshold, for patient 5. (b) CT and PET‐defined target volumes for patient 5. The comp_GTV (CT) volume is shown in violet and the ITV in blue. The PET‐defined volume, based on a 15%‐of‐maximum segmentation threshold, is depicted in the yellow “wire mesh.”

Figure 1(a) shows an example comp_GTV (CT‐defined) volume (left) and the corresponding PET‐defined target generated using a 20%‐of‐maximum segmentation threshold (right) for patient 4. A digitally reconstructed radiograph of the superimposed volumes is illustrated (for patient 4) in Fig. 1(b). The comp_GTV (CT) is shown in violet and the ITV in blue; the PET‐defined volume, based on a 15%‐of‐maximum segmentation threshold, is depicted in the yellow “wire mesh.”

### B. Monte Carlo‐based treatment planning and analyses

Treatment planning for all patients was performed using the Dose Planning Method^(18)^ Monte Carlo code integrated within the UMPlan radiotherapy planning software (RT_DPM^(19)^). RT_DPM has been previously benchmarked against measurements in water and heterogeneous phantoms and has been found to be accurate (within 1% to 2% of measurements) even under conditions of lateral electronic disequilibrium.^(19)^ The Monte Carlo method is known to be the most accurate dose calculation algorithm in inhomogeneous tissues, such as the lung, and as such increases our confidence in the doses delivered to targets and normal lung tissues in this study. Treatment plans were generated to produce dose uniformity of approximately 100±5% within the ITV, to ensure optimal dose coverage to the comp_GTV. Dosimetric analyses were subsequently performed for the PET‐defined targets by evaluating doses that the targets and normal lung tissues would have received in the ITV‐designed treatment plan. The following dose metrics and indices were evaluated: dose volume histograms (DVHs), generalized equivalent uniform dose (gEUD) for the respective target volume, mean lung dose (for the normal lung), and dose for the PET target to produce the same normal tissue complication probability (NTCP) as that for the composite CT GTV. Normal lung tissue was defined by subtracting the respective GTV from the combined lung volume. The gEUD, introduced by Niemierko,^(20)^ is defined as the uniform dose distribution that gives an effect equivalent to that of a given heterogeneous dose distribution. It can be derived from DVHs using a generalized mean dose, with $v_{i}$ being the fractional volume receiving a uniform dose $D_{i}$:

(1) $$\text{EUD}={\left(\sum_{i=1}^{N}v_{i}D_{i}^{a}\right)}^{\frac{1}{a}}$$

*N* is the number of voxels in the target, $D_{i}$ the dose in voxel *i,* and *a* the dose‐volume effect parameter specific to the structure of interest. In this study, gEUD for the target was calculated using a=−10, a value representative of tumors.^(21)^ NTCP was calculated using the Lyman model^(22)^ based on the effective volume DVH reduction methodology described by Kutcher and Burman.^(23)^ Lyman's model uses a normal probability distribution function to describe the sigmoidal shape on the dose response for an organ. The relevant model parameters for the normal lung, TD(50)=30.8Gy at 2.0 Gy per fraction, m=0.99, and n=0.37, were acquired from a previous study.^(24)^

Monte Carlo treatment planning was carried out using cubic voxels (side=5mm), a 2‐mm step size, and low‐energy electron and photon cut‐off values of 200 keV and 50 keV, respectively. For each treatment plan, approximately 1 to 1.5 billion histories were simulated, resulting in 1 σ statistics of less than 1% in calculated dose on average within the ITV.

## III. RESULTS AND DISCUSSION

Treatment plans for eight patients were analyzed in terms of the biological dose indices, gEUD for the target, and NTCP and mean lung dose to the normal lung tissue. Although these indices are limited in their applicability to individual patient situations, they provide a reasonable measure of the target dose homogeneity (in the case of gEUD), as well as an assessment of “safety” in terms of doses delivered to the normal lung tissue (with respect to NTCP). As a result, they are often used in radiotherapy treatment planning as gauges of the efficacy of a plan in optimizing the therapeutic ratio.

### A. Target dose analysis

Table 1 shows tumor locations and volumes defined by CT (comp_GTV) and PET for all eight patients. PET‐defined volumes are reported for threshold values ranging from 20% to 50% of the maximum intensity pixel. As expected, the volume decreases as the segmentation threshold increases. Substantial differences are sometimes noted in the PET‐defined target volumes as a function of segmentation threshold. For example, the target volume for patient 3 is reduced by 35% in changing from a 15% to a 20% segmentation threshold. A detailed analysis of the PET‐defined target volumes at various thresholds is provided by Fernando et al.^(17)^

**Table 1:** Table of tumor locations and volumes defined by CT (comp_GTV) and PET. PET‐defined volumes are reported for threshold values ranging from 15% to 50% of the maximum intensity pixel. Note that the 15% threshold volume was not delineated for all patients. For the tumor locations, example abbreviations are as follows: RUL=right upper lobe,LLL=left lower lobe.

Tumor location and volume (in ${\text{cm}}^{3}$)	Patient #1	Patient #2	Patient #3	Patient # 4	Patient #5	Patient #6	Patient #7	Patient #8
Location	RUL	RUL	LUL	R hilar	RLL	RLL	RLL	LLL
comp_GTV CT	876.2	471.5	172.4	411.2	34.5	301.5	82.2	134.2
PET_15%	—	426.5	—	—	—	258.4	64.7	—
PET_20%	953.8	274.2	286.2	438.6	46.4	209.9	46.8	234.7
PET_30%	783.9	151.1	164.5	266.8	25.9	155.1	30.2	125.7
PET_40%	647.0	85.4	118.0	224.4	16.6	126.1	21.7	86.4
PET_50%	488.3	53.8	82.9	184.5	10.4	101.0	15.3	52.2

In Table 2, gEUDs are reported for the CT (comp_GTV) and PET‐defined target volumes for eight different patient treatment plans. Each plan was generated using the CT‐defined ITV to ensure adequate dose coverage (within approximately ±5%) to the comp_GTV; the prescribed doses are presented in bold. Values in parentheses correspond to the dose differences (in gray) between the gEUDs for the given PET target and the comp_GTVs in each patient. It is generally found that the gEUD differences for the PET‐defined targets are reduced as the segmentation threshold increases from 10% to 50% of the maximum intensity. This is because the target volume decreases as the threshold increases: a smaller target will receive a higher dose and will thereby maintain a higher gEUD. Despite the large variation in the target volumes (shown for example for patient 2 in Fig. 2(a)), only minor differences were noted in the gEUDs between the various volumes in patients 1, 2, and 5 to 7. Although maximum differences of up to 1.6 Gy were found (in patient 5, e.g.), these are unlikely to be clinically meaningful because they are less than one (2.0 Gy) fraction. The minor gEUD differences are primarily due to the large comp_GTVs in these plans, which ensures coverage of the 95% isodose line (IDL) for all target volumes. This is illustrated in Fig. 2(a), where we find that due to the large comp_GTV, even the largest PET target (PET_10%) is encompassed by the 95% IDL; as a result, the target gEUDs are uncompromised. DVHs for the CT and PET‐defined targets are shown in Fig. 2(b) for patient 2. The subtle variation among the DVHs corroborates the small changes in gEUD in Table 2 for this plan.

**Table 2:** Table of generalized equivalent uniform dose (gEUD) in gray for the CT‐ (comp_GTV) and PET‐defined target volumes for eight different patient plans. PET targets were delineated using percentage thresholds of the maximum intensities; thresholds ranged from 10% to 50%. Note that EUDs were not evaluated for all PET‐defined volumes; undefined values are indicated with a dash. Doses (in Gy) prescribed to the comp_GTV (within approximately 100±5%) are shown in bold. Values in parentheses correspond to the dose differences (in grays) between the gEUDs for the given target and the comp_GTVs in each plan.

Target	Patient #1	Patient #2	Patient #3	Patient #4	Patient #5	Patient #6	Patient#7	Patient#8
	45 Gy	60 Gy	62 Gy	60 Gy	62 Gy	56 Gy	74 Gy	60 Gy
comp_GTV	44.4	5 8.9	62.1	59.3	61.4	54.4	73.2	58.9
	(0.0)	(0.0)	(0.0)	(0.0)	(0.0)	(0.0)	(0.0)	(0.0)
PET_10%	44.4	58.4				54.6		
	(0.0)	(−0.4)	—	—	—	(+0.2)	—	—
PET_15%	44.5	58.9		55.2	60.1	54.4		6.7
	(+0.1)	(0.0)	—	(−4.1)	(−1.3)	(+0.0)	—	(−52.2)
PET_20%	44.5	58.9	56.3	55.4	59.8		73.4	3.9
	(+0.1)	(0–0)	(−5.7)	(−3.9)	(−1.6)		(+0.2)	(−55.0)
PET_25%			59.7		60.6	53.9		
	—	—	(−2.3)	—	(−0.8)	(−0.5)	—	—
PET_30%	44.5	58.9	61.5	54.2	61.0	53.6	73.4	2.7
	(+0.1)	(0.0)	(−0.5)	(−5.1)	(−0.4)	(−0.8)	(+0.2)	(−56.2)
PET_40%	44.5	58.8	61.5	54.7	61.6	53.4	73.3	2.7
	(+0.1)	(−0.1)	(−0.5)	(−4.6)	(+0.2)	(−1.0)	(+0.1)	(−56.2)
PET_50%	44.5	5 8.9	61.3	55.6	62.0	53.1	73.3	4.3
	(+0.1)	(0.0)	(−0.7)	(−3.7)	(+0.6)	(−1.3)	(+0.1)	(−54.6)

**Figure 2(a):**
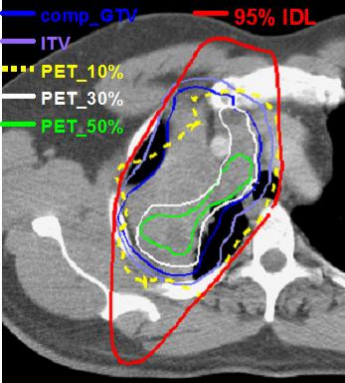
PET and CT (comp_GTV and ITV) target volumes on an axial cut for patient 2. PET volumes are presented for segmentation thresholds of 10%, 30%, and 50% of the maximum intensity. The plan was designed to produce a dose uniformity of 100±5% within the ITV to ensure optimal dose coverage to the comp_GTV. The 95% isodose line (IDL) is included to illustrate the dose coverage to all targets.

**Figure 2(b):**
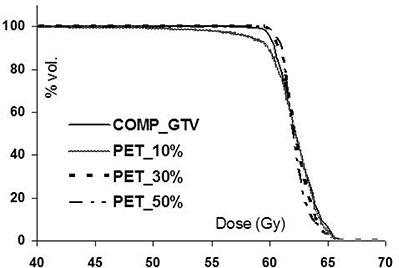
Dose‐volume histograms for patient 2 shown for the comp_GTV and PET volumes delineated using 10%, 30%, and 50%‐of‐maximum segmentation thresholds.

For patients 3, 4, and 8, substantial variation is found in the gEUDs among the various targets. The gEUD differences (up to ~6.0Gy in patients 3 and 4) are due to the extension of the PET‐defined targets outside the ITV resulting in underdosage to these targets. Patient 8 represents an extreme situation in which doses to the PET‐defined targets are significantly compromised as a result of their considerable extension outside the ITV. Target volumes for patient 8 are shown in Fig. 3(a), where significant underdosage of all PET‐defined targets is noted. Regions of all PET targets fall outside the 95% or lower IDLs, which produces a drastic reduction in the gEUD, as noted in Table 2 for patient 8. Figure 3(b) shows target DVHs for patient 8, where the PET‐defined target dose reduction is clearly evident. It should be pointed out that the target gEUD differences in patients 3, 4, and 8 (see Table 1) are likely to be clinically relevant because in some cases they are much greater than a single 2.0 Gy fraction.^(25)^ If we average values over all patients, we find that in comparison to the comp_GTVs, PET target gEUDs differ by −12.7%(σ=29.3%) with a 95% confidence interval (CI) of −4.2 to −21.1%.

**Figure 3(a):**
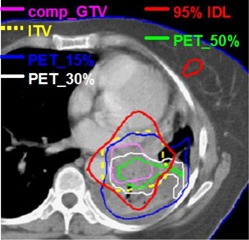
PET and CT (comp_GTV and ITV) target volumes on an axial cut for patient 8. PET volumes are presented for segmentation thresholds of 15%, 30%, and 50% of the maximum intensity. The plan was designed to produce a dose uniformity of 100±5% within the ITV to ensure optimal dose coverage to the comp_GTV. The 95% isodose line (IDL) is included to illustrate the dose coverage to all targets.

**Figure 3(b):**
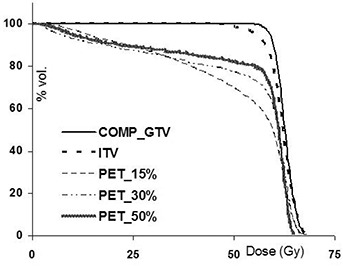
Dose‐volume histograms for patient 8 for the comp_GTV and PET volumes delineated using 15%, 30%, and 50%‐of‐maximum segmentation thresholds.

### B. Normal lung tissue dose analysis

Shown in Table 3 are mean lung doses (MLDs) for all patients evaluated. Normal lung volumes were defined by subtracting the given target volume from the combined (left+right) lung volume. The variation in MLDs among the normal lung tissues calculated using CT and PET‐defined targets is small: maximum differences are on the order of 1 Gy, suggesting that the MLD is relatively insensitive to even large changes in the target volume. This is not unexpected considering that the target volume is usually much smaller than the combined lung volume. The mean variation in MLD relative to the comp_GTV (averaged over all patients) is 1.1% (σ=4.2%), with a 95% CI of −2.3 to −0.1%. It is also seen that as the PET‐defined target volume decreases (with increasing threshold), the MLD increases because the volume of irradiated normal lung (lungs ‐ target) increases. Recall also that treatment plans were designed for optimal dose coverage to the comp_GTV. Significant differences are observed in the doses to restore the NTCP for normal lung using the comp_GTV volumes in patients 1, 2, 4, and 6. For example, in patient 2, an additional 8.7 Gy (corresponding to 68.7 Gy) may be delivered to the PET_10% volume relative to the comp_GTV for equivalent NTCPs. This occurs because the PET_10% volume is larger than the comp_GTV; the corresponding irradiated normal lung volume is therefore smaller than that of the comp_GTV, resulting in a smaller MLD and NTCP. Consequently, the dose to the PET_10% target is increased relative to that of the comp_GTV for an equivalent normal lung NTCP. Note that this effect is reversed as the PET target volume becomes smaller than that of the comp_GTV; the dose to the PET_50% target, for example (in patient 2), decreases by 5.8 Gy (corresponding to 54.2 Gy) relative to the comp_GTV.

**Table 3:** Table of mean lung dose in gray for the normal lung tissue for eight different patient plans. Normal lung tissue was defined by subtracting the given target volume from the combined lung volume. Doses (in gray) prescribed to the comp_GTV (within approximately 100±5%) are shown in bold. Values in parentheses correspond to the dose differences (in gray) between the comp_GTV prescribed doses and the doses delivered to the given target to produce the same normal lung tissue effect (NTCP). For example, in patient 4, the dose delivered to the PET_20% target to produce the same NTCP as for the comp_GTV is +3Gy greater than the comp_GTV prescribed dose (or 63 Gy). Note that normal tissue dose indices were not evaluated for all PET‐defined volumes; undefined values are indicated with a dash.

Normal lung tissue	Patient #1	Patient #2	Patient #3	Patient #4	Patient #5	Patient #6	Patient #7	Patient #8
	45 Gy	60 Gy	62 Gy	60 Gy	62 Gy	56 Gy	74 Gy	60 Gy
lungs ‐comp_GTV	24.7	9.8	6.0	18.0	5.7	6.7	6.4	14.4
	(0.0)	(0.0)	(0.0)	(0.0)	(0.0)	(0.0)	(0.0)	(0.0)
lungs‐PET_10%	22.9	8.6				6.5		
	(+3.7)	(+8.7)	—	—	—	(+1.7)	—	—
lungs‐PET_15%	23.9	9.9		18.0	5.6	6.9		14.1
	(+1.5)	(−0.5)	—	(+0.2)	(+1.7)	(−1.3)	—	(+1.6)
lungs‐PET_20%	24.2	10.4	6.0	18.2	5.6	7.0	6.6	14.2
	(+1.1)	(−3.4)	(+0.6)	(+3.0)	(+0.8)	(−2.6)	(−1.3)	(+0.8)
lungs‐PET_25%			6.0		5.7			
	—	—	(0.0)	—	(−0.1)	—	—	—
lungs‐PET_30%	24.5	10.7	6.0	18.3	5.8	7.1	6.6	14.4
	(+0.3)	(−5.0)	(0.0)	(−1.1)	(−0.8)	(−3.4)	(−1.6)	(+0.3)
lungs‐PET_40%	24.7	10.8	6.0	18.4	5.8	7.2	6.6	14.4
	(+0.1)	(−5.6)	(0.0)	(−1.2)	(−1.4)	(−3.8)	(−1.6)	(−0.1)
lungs‐PET_50%	24.7	10.8	6.0	18.4	5.9	7.3	6.6	14.5
	(0.0)	(−5.8)	(0.0)	(−1.2)	(−1.7)	(−4.5)	(−1.7)	(−0.2)

**Figure 4:**
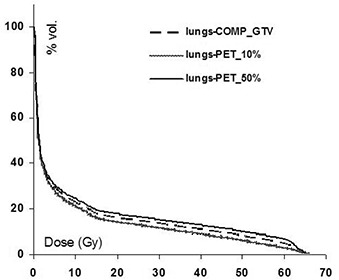
Dose‐volume histograms for patient 2 for the normal lung tissue defined by subtracting the target volume from the combined (left+right) lung volume.

**Figure 5:**
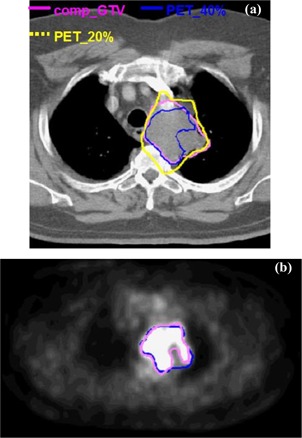
(a). Variation of PET and CT targets for patient 3 showing minimal overlap of the target volume into the normal lung tissue. Contours are shown for the comp_GTV (CT‐based) and PET‐defined volumes based on 20%‐ and 40%‐of‐maximum segmentation thresholds.(b). PET image for patient 3 showing the region of tumor 18‐FDG uptake. The comp_GTV (CT‐defined) and the PET‐defined target (generated using a 25%‐of‐maximum segmentation threshold) are illustrated in the blue and violet contours, respectively.

DVHs for normal lung tissue are presented in Fig. 4 for patient 2. Although both the DVH (and NTCP) differences in the normal lung defined using CT and PET targets are relatively small, the prescribed dose in this particular case falls along the high gradient region of the dose‐effect curve. As a result, even a small change in NTCP causes a large change in the dose to restore the comp_GTV NTCP, as seen in Table 3. Variation in the MLDs and doses to restore the comp_GTV NTCP are markedly reduced for patient 3 in comparison to all other patients. Upon closer observation, it was revealed that the location of the tumor in patient 3 (see Fig. 5) excluded most of the normal lung tissue; the differences in the PET volumes and the comp_GTV occur primarily within the tumor (Fig. 5(a), (b)). The impact on the normal lung volumes, and accordingly the MLD and NTCP, is therefore insignificant.

## IV. CONCLUSION

For the patients investigated in this study we found that the variation in PET‐defined target volumes delineated using different segmentation thresholds can result in significant and clinically important dose differences to targets and normal tissues. Accurate target segmentation with PET is therefore critical for accurate dosimetry and should be carefully assessed before PET volumes can be used faithfully in treatment planning. Although target segmentation is confounded by spatial resolution issues, validation of PET volumes may be performed using CT volumes from datasets that incorporate the extents of breathing motion. In this regard, recent efforts to quantify motion effects and the ability to sort PET images according to breathing phase (4D PET^(^ ^26^ ^,^ ^27^ ^)^) are useful in reducing uncertainties associated with organ segmentation. Another important concern with the use of PET in conjunction with CT is the image registration process. Since the PET and CT datasets were acquired on different scanners in this study, factors such as variation in patient positioning may have negatively impacted the image registration. Moreover, the use of local versus global measures in the registration process and their impact on dose requires further investigation. Further study on the correlation of outcome (local control and normal tissue toxicity) with target volume is necessary to assess the utility of PET‐defined targets in radiotherapy treatment planning. Finally, the use of the Monte Carlo method has allowed us to accurately assess the influence of PET‐defined target volume variation on doses to the targets and normal tissues; that is, the results are not influenced by limitations in the dose calculation algorithm as they may well be with the use of non‐Monte Carlo‐based algorithms for lung cancer treatment planning.

## ACKNOWLEDGMENTS

We congratulate Dr. Greenfield on his 90th birthday, and we thank him for his pioneering efforts in the field of medical physics. We wish to honor Dr. Greenfield's lifetime of achievement and to dedicate this paper to the memory of Dr. Edward J. Hoffman. Dr. Hoffman was the co‐inventor of PET, the director of the UCLA Biomedical Physics graduate program (founded by Dr. Greenfield), and was a mentor to author IJC. Dr. Hoffman was a wonderful human being whose presence is greatly missed by all those who knew him.

This work has been supported in part by grants NIH P01‐CA59827 and R01 CA106770.
